# Effect of *Populus nigra* spring and autumn leaves extract on *Capsicum annuum* infected with pepper mild mottle virus

**DOI:** 10.1038/s41598-022-24786-2

**Published:** 2022-12-23

**Authors:** H. A. Gharib, A. M. Mandour

**Affiliations:** 1 Timber Trees and Forestry Research Department, Horticulture Research Institute, Agricultural Research Center, 9 St. Al-Gamaa, Giza, Egypt; 2 Plant Virus and Phytoplasma Research Department, Plant Pathology Research Institute, Agricultural Research Center, 9 St. Al-Gamaa, Giza, Egypt

**Keywords:** Physiology, Plant sciences

## Abstract

*Capsicum annuum* is one of the main vegetable crops for the local market and exportation in Egypt. In this concern, pepper mild mottle virus (PMMoV) infection caused a significant decrease in *Capsicum sp.* leading to large economic losses. An isolate of PMMoV was obtained from naturally infected pepper plants, exhibiting different patterns of mottling, leaf distortion, yellowing, and stunting of leaves. The virus was identified. The molecular detection of PMMoV was done using RT-PCR with specific primers designed for coat protein genes. An RT-PCR product (474) bp of the coat protein gene of (PMMoV) was cloned. The target of the investigation was the effect of spring and autumn ethanol extracts of *Populus nigra* leaves on *C. annuum* seedling growth and infected *C. annuum* with (PMMoV) under greenhouse conditions. The experimental data showed that treated spring leaf extract of *P. nigra* enhanced infected *C. annuum* seedling growth parameters and fruit quality compared to uninfected seedlings. *P. nigra* spring leaf extract containing some allo-chemicals had a negative effect on uninfected seedlings. *P. nigra* autumn leaf extract significantly improved the growth and fruit quality of infected *C. annuum* seedlings compared to the control.

## Introduction

*Populus nigra* known as cottonwood, poplar and aspen deciduous trees^1^. Trees produce a large quantity of fallen leaves as a waste during autumn in Egypt ‘s environment. *P. nigra* is a member of the Salicaceae family, which included several species and had distributed extensively throughout the world^2^. Poplar leaves used as a boost to antimicrobials^3^. A lot of bioactive structures, such as terpenoids and flavonoids, far more to phenolic compounds, have been extracted from *Populus sp*. by^4–7^. On this side, the results provided a hopeful baseline in sequence for using flavonoids from these trees as antimicrobials to control plant diseases^7^. Meanwhile, different flavonoids with the structure pinobanksin and 3,7-dimethylquercetin as well as pinocembrin separated from the *P. nigra* ethanol extract^8^.

On the other side, phenolic structures with caffeic and p-coumaric additions to cinnamic were mentioned^6^. Previous investigations studied poplar trees’ naturally occurring aromatic compounds, such as salicylic acid and salicylic alcohol^9,10^. Plants and their extracts used for medicinal purposes since ancient times. According to the World Health Organization, over 75–80 percent of the world’s population uses plant medicine in some form or another. Willow bark, for example, eaten by ancient Egyptians to ease fevers and headaches. Scientists found thousands of years later that the bark contained salicylic acid, the key element in aspirin. John Buckner discovered salicyl alcohol glucoside (Salicine) from willow bark in 1928, and Raffaele Piria called the compound salicylic acid (hereinafter SA) in 1938^11^.

Sweet pepper *C. annuum* is a member of the solanaceae family of vegetables. It had considered a major greenhouse yield cultivated during different seasons to meet increasing demand in Egypt. The cultivation area reached 91,404 feddan^12^.

Pepper mild mottle virus (PMMoV) has only recently identified on commercial bell pepper fields in Florida, Italy,^13^. The virus had spread by mechanical means and infected seeds but cannot transmit by insects. It has grown worldwide in field-grown bell, hot, and ornamental peppers. It found in pepper cultivars where production practises are typical for the rapid spread of the disease^14^.

As foliar symptoms can be mild, infected plants may did not notice till fruit symptoms were evidently resulting in spread to adjoining plants and higher yield losses^13^. (PMMoV) causes serious economic losses in pepper production in China. ^15^ identified two PMMoV isolates (named PMMoV-ZJ1 and PMMoV-ZJ2) with decrement symptoms in a survey for viral diseases on pepper in Zhejiang province. (PMMoV) infected fruits general appeared small and malformed and this was obvious by off-colored sunken areas. ^16^ found that a virus was causing damage to pepper yield.

The main target of this demonstration evaluated leaves of *Populus nigra* as a natural woody trees products had a chemical values. In this concern applied to study the effect of foliar (25–50 and 100%) concentrations of spring and autumn leaves (APLE) on *C. annuum* seedlings and fruit parameters. As well as studying the effects of (25–50 and 100%) concentrations of (SPLE) and (APLE) foliar applications, the process took 20 and 40 days from germinating infected pepper under greenhouse conditions in the Ismailia region.

## Material and methods

This investigated study highlight the important of *Populus nigra* leaves and indicator differences between chemical strictures during spring and autumn content. In this concern, clear new untraditional process struggle PMMoV infections. During the spring on 2020, the current trial had carried out at Ismailia Governorate, Egypt. After pepper seedlings had three or four true leaves transplanting, *Capsicum annuum* was cultivated on pots 30 cm in mixed loam and sand (1:1) under greenhouse conditions. Mineral fertilizer had using the nitrogen fertilizer in the structure of calcium nitrate (17% N), potassium fertilizers in the structure of potassium sulphate (48% K_2_O) and phosphorus fertilizers in the structure of phosphoric acid (61.5% P_2_O_5_) according to Table 1.To investigate the effect of concentrations (% 25–50 and 100) foliar application of spring *Populus nigra* leaves extract (SPLE) and autumn *Populus* leaves extract (APLE) individuals after (20 and 40 days) respectively pepper transplant on growth parameters without infection as a split block treatment. On this concern pepper plant had infected with (PMMoV) during a first week after transplant, then treated at (20 and 40 days) respectively from transplant with foliar application extract, form (SPLE) and (APLE) individual as another split block treatment.

**Table 1:** Fertilizer program of *Capsicum annuum* under greenhouse conditions (g/pot).

Week after transplanting	Calcium nitrate	Potassium sulphate	Phosphoric acid
2	0.15	0.10	0.02
3	0.35	0.20	0.05
4	0.50	0.30	0.08
5	0.70	0.40	0.10
6	1.00	0.60	0.15
7	1.00	0.60	0.15
8	1.00	0.60	0.15
9	1.00	0.60	0.15
10	0.70	0.40	0.10
11	0.70	0.40	0.10
12	0.70	0.40	0.10
13	0.70	0.40	0.10

### Greenhouse conditions

Greenhouse air was 21–30 °C during daylight and 16–18 °C at nighttime. Atmosphere had relative humidity from 40 to 90%. The suggested vapor pressure deficit (VPD) might be from 3 to 7 g/m^3^^17–20^. The solar radiation between 200 and 450 W/m^2^ within the gable-even-span greenhouse was 6 measured and evidenced for short and long requisites. In contrast, other greenhouses conditions control using some apparatus, like black net sheets and natural airing systems^20^.

### Tree materials

Collection of tree material poplar tree spring leaf collected in March 2019 and 2020 from trees growing in the nursery of timber trees department at the Horticulture Research Institute Agriculture Research Center, Giza, Egypt. Senescent leaves were collected in September (2018 and 2019). The samples dried in the electric oven at 40 °C until they reached constant weight, according to^21^. Dried material was ground by an electric mixer to find the crush forms of each sample. The powder preserved in sterilised glass jars.

### Preparation extracts samples

Then samples were air dried in the laboratory for seven days under room conditions and later in the electric oven for two days at 40 °C^22^. The dried material then pulverised using a blender (electric mixer) to get powder forms of each sample. The powder collected and kept in clean and sterile conditions. Each leaf in spring and autumn, an individual dried powdered sample 500 g had lain in a 2000 ml beaker and processed by drenching 1000 ml of ethanol solvent. Then they enclosed with aluminium foil and put into a water bath 60 °C and had shaken to get homogenous solutions. After that, the samples filtered and evaporated by a rotary evaporator 60 °C to isolate the solvent extract and store it in clean-capped glass bottles and reserve it in the refrigerator for reuse^23^.

### Source of virus isolates

**Figure 1:**
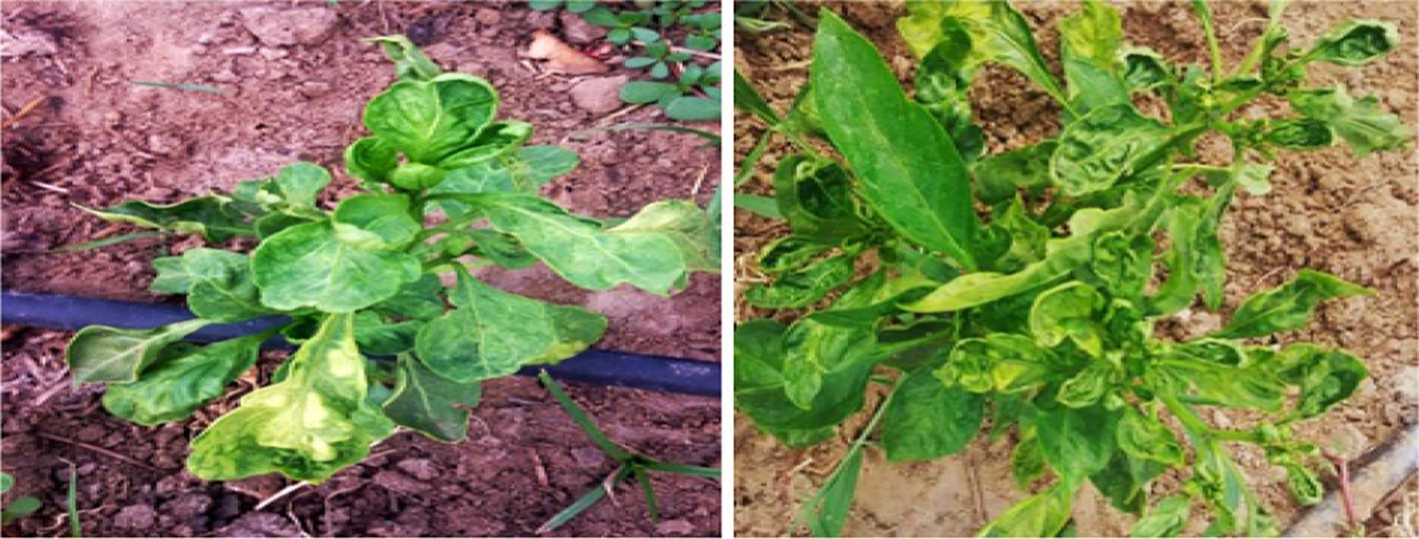
The naturally infected pepper plant had indicator symptoms including mottling, leaf distortion, and stunting in the field.

Several field visits had been conducting to pepper plant growing areas in the Ismailia Governorate. The naturally infected pepper plants contain viral symptoms including mottling, leaf distortion, yellowing, and stunting collected according to Fig. 1. After being collected from the field, the infected leaf samples had placed in cool boxes and stored at 80 °C for later use.

### Mechanical inoculation

Mechanical inoculation carried out according^24^. Infectious sap extracted from young leaves indicator clear and typical symptoms. Leaves were ground in a sterilized mortar with a few drops of 0.2 M phosphate buffer. The extracted sap filtrated through two layers of chasse cloth and then centrifuged for 5 min. at 5000 rpm. Before it was used to inoculate the leaves of tested plant seedlings. The infected plant extract measured on a NanoDrop device and found that the virus concentration was high.

### Propagation of virus isolates

Infected leaf samples were ground in a phosphate buffer solution (pH7.2). Infectious sap had mechanically inoculated onto *Chenopodium amaranticolor*. The single local lesion assay used for biological purification of the isolate and propagated on healthy pepper plants.

### Virus identification

#### Host range and symptomatology

Twenty-one plant species belonging to four families mechanically inoculated with infectious crude sap expressed from pepper plants. The seedlings of each host species inoculated and observed daily for symptom development, and the mechanically inoculated plants kept under observation in insect-proof cages under the greenhouse. Three weeks later, plants examined visually for any signs of symptom appearance. Symptomless plants were checked for virus infection by back inoculation of *Chanopodium amaranticolor* leaves and/or the ELISA technique by^25^.

**Figure 2:**
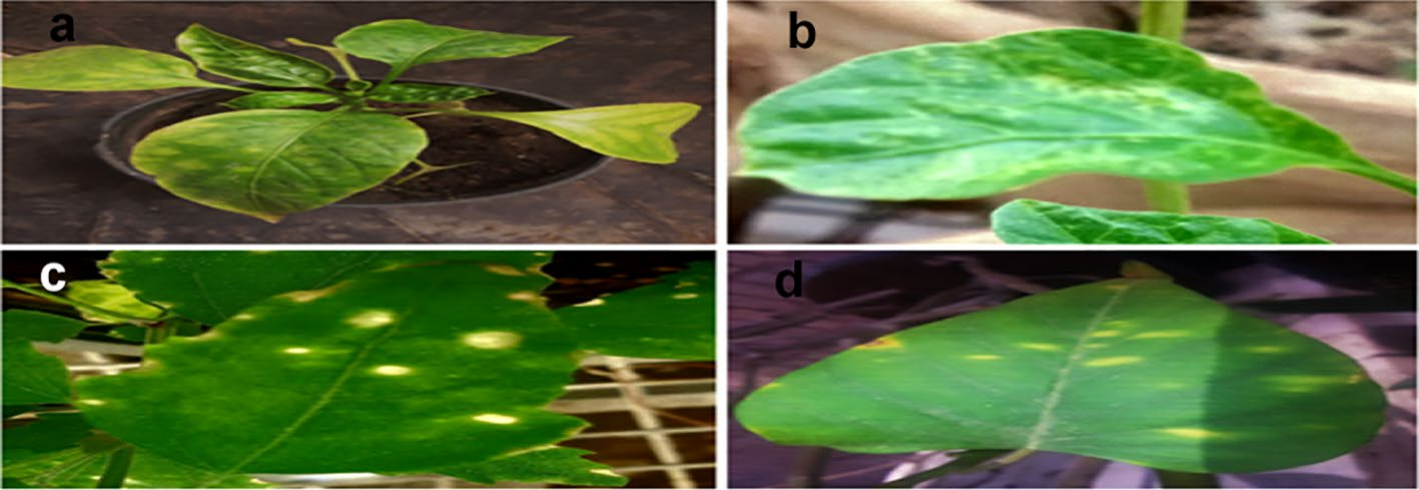
Symptomology of PMMoV (**a**) mottling and yellowing on *Capsicum annum* L. cultivar California, (**b**) mottling on *Capsicum fratescens* L. cv. Chilli (**c**) Local chlorotic lesions on *Chanopodium amaranticolor* (**d**) Local necrotic lesions on *N. glutinosa.*

Different symptoms observed on the infected pepper plants. This virus infection had indicator mottling, leaf distortion, yellowing, and stunting. This virus propagated on pepper plants, which developed the same symptoms as those in naturally infected plants. This isolated of PMMoV indicator different styles of symptoms on pepper hosts, such as mild mottling, mottling, yellowing, and malformation (Table 1 and Fig. 2). The incidence of PMMoV confirmed by back inoculation with *Chanopodium amaranticolor*. The tested plants could divide, according to their reactions, into two groups:

#### Susceptible hosts to PMMoV

##### Plants reacted with systemic symptoms

Systemic symptoms observed in the tested *Capsicum annum* L. cv. California, *Capsicum fratescens* L. cv. Chilli and *Nicotiana clevelandii* Fig. 2. Systemic symptoms, general appear nearly 11–14 days after inoculation.

##### Plants reacted with local lesions

Virus isolate produced chlorotic local lesions on the inoculated leaves of *Chanopodium amaranticolor*, Ch. quinoa and necrotic local lesions on the inoculated leaves of *Datura metal*, *Datura stramonium*, *Nicotiana tabacum,* and *N. glutinosa* nearly 7–10 days after inoculation (Fig. 2).

#### Unsusceptible plants

These plant species were not susceptible to pepper infection. These plants belong to different families: Cucurbitaceae, Fabaceae, and *N. arusica*. Host range studies for diagnosis will usually be most useful for those infecting a relatively narrow range of plants^26^.

The general outlook of the result in Table 2 indicator that the studied isolate of PMMoV had a wide host range between members of the family Solanaceae. On the other side, the virus infects a few species of Chenopodiaceae. PMMoV induced mottling, yellowing, and malformation symptoms in the family Solanaceae. The informed data in Table 1 confirmed the results of^27^.

**Table 2:** The reaction of different hosts to Pepper mild mottle virus.

Family	Host plant	Symptoms
*Chenopodiaceae*	*Ch. amaranticolor* Coste and Reyn *Ch. quinoa* Wild *Beta vulgaris*	CLL CLL NS
*Cucurbitaceae*	*Cucurbita pepo* cv. Cavili *Cucurbita pepo* cv. Eskandarni *Cu. maxima* cv. Wintersquash *Cucumis sativus* cv. Balady *Citrullus lanatus* cv. Giza 2	NS NS NS NS NS
*Fabaceae*	*Glycine max* L.cv.Giza22 *Lupinus termis* cv. Lupine *Phaseolus vulgaris* cv. Giza 4 *Pisium sativum* L. cv. Sugar sweet *Vicia faba* cv. Giza 3	NS NS NS NS NS
*Solanaceae*	*Capsicum annum* L. cv. California *Capsicum fratescens* L.cv. Chilli *Datura metal* *Datura stramonium* *Nicotiana tabacum* L.cv.Whit Burley	M + Y M + Mf NLL NLL NLL

### Modes of transmission

#### Mechanical transmission

Inoculums prepared by homogenising infected pepper leaves with a few drops of phosphate buffer (pH 7.2) in a sterilised mortar. Leaves of host plants previously dusted with carborundum (600 mech) rubbed with the forefinger or with a cheesecloth pad previously soaked in the inoculum. The plants rinsed with tap water and kept in the insect proof greenhouse. Obtainment results revealed that PMMoV easily transmitted mechanically to indicator hosts like *Chenopodium amaranticolor* which indicator chlorotic local lesions.

#### Insect transmission

Two aphid species, name, *Aphis faba* (scop) and *Myzus persicae* (sulz) checked for their ability to transmit the isolated virus. *A. faba* (scop) and *M. persicae* (sulz) maintained on virus- free health faba beans for *A. faba* (scop) and cabbage plants for *M. persicae* (sulz) and kept under insect-proof cages in the greenhouse. The aphids starved for one hour and then transferred to feeding for a 30 min acquisition feeding period on diseased pepper plants. At the end of the feeding period, aphids transferred to healthy plants at a rate of 10 aphids/plant. After a 24 h feeding period, the insects had killed by spraying all tested plants with an effective insecticide (malathion 0.2%). Symptoms and the percentage of transmission recorded.

Results indicator these *A. faba* (scop) and *M. persicae* (sulz) did not able to transmit the virus. None of the tested plants produced any symptoms.

### Seed transmission of virus

To study the transmission of (PMMoV) through seeds. Two hundred pepper seeds cv. California collected from previously inoculated infected peppers had sown in 20 cm sterilized pots and kept in an insect- proof greenhouse for symptom observation for three weeks after sowing, and the percentage of seed transmission calculated. (PMMoV) transmitted through pepper seeds. Data showed that the percentage of seed transmission differed according to cultivar. (PMMoV) transmitted at 38%. The result confirmed using ELISA.

### Molecular characterization

#### RNA extraction

RNA extraction from leaf samples carried out using the RNeasy Plant Mini Kit (QIAGEN) according to the manufacturers’ instructions.

#### Primers for the coat protein gene of (PMMoV)

For the amplification of the capsid protein (CP) gene (474 bp), two pairs of specific primers (CP/s: 5′-ATGGCATACACAGTTACCAGT-3′) and (CP/a: 5′-TTAAGGAGTTGTAGCCACACGTA3′) used in RT-PCR^28^.

#### One-step RT-PCR

One-step RT-PCR reactions carried out using the “iScript One Step qRT-PCR Kit” (BIOMATIK) in a 25 µL reaction volume. Each reaction contained 1 µL of the RNA extract (40 mg of total RNA), 12.5 µLi Green Mastermix, 1.5 µL of 10 µM of each primer, 0.5 µL of qRT-PCR Enzyme Mix, and 25 μL of nuclease-free water. Synthesis of cDNA done at 42 °C for 30 min and denaturation at 95 °C for 10 min, followed by 35 cycles of 94 °C for 30 s, 50 °C for 1 min, 72 °C for 1 min, and a final cycle of 72 °C for 10 min (Velasco et al. 2011). 5 μL of PCR products were loaded into 1% agarose gels with a 100 bp DNA ladder (BIOMATIK) and pictures taken under UV light with a digital imaging system gel doc (Syngene Bio Imagins, IN Genius).

#### Analysis of RT-PCR products

**Figure 3:**
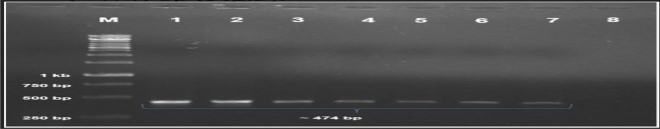
Agarose gel electrophoresis of RT-PCR amplified products. M: 1 kb DNA ladder (Promega); 1–7: seven infected pepper samples; 8: a negative sample.

The *cp* genes of PMMoV collected from Ismailia had isolated using RT-PCR with specific primers. The PMMoV-*cp* gene had (~ 474) bp as shown in (Fig. 3).

#### Experimental design and treatments

A randomized complete block design used with fourteen treatments, everyone had sixteen plants, including control. It had contained four replicated. Each one contained four pots had one plant per pot. Pepper seedlings treated with extract by spraying whole leaves, even run-off, with different (PLE) concentrations after 20 and 40 days, respectively, follows:

1. Pepper seedling untreated control.
2. Pepper seedlings had foliar with 25% (SPLE) + 75% tap water.
3. Pepper seedlings had foliar with 50% (SPLE) + 50% tap water.
4. Pepper seedlings had foliar with 100% (SPE).
5. Pepper seedlings had foliar with 25% (APLE) + 75% tap water.
6. Pepper seedlings had foliar with 50% (APLE) + 50% tap water
7. Pepper seedlings had foliar with 100% (APLE).
8. Pepper seedling infected (PMMoV) without foliar application.
9. Pepper seedlings infected (PMMoV) foliar with treated with 25% (SPLE) + 75% tap water.
10. Pepper seedlings infected (PMMoV) foliar with 50% (SPLE) + 50% tap water.
11. Pepper seedlings infected (PMMoV) foliar with 100% (SPLE).
12. Pepper seedlings infected (PMMoV) foliar with 25% (APLE) + 75% tap water.
13. Pepper seedlings infected (PMMoV) foliar with 50% (APLE) + 50% tap water.
14. Pepper seedlings infected (PMMoV) foliar with 100% (APLE).

#### Gas chromatography (GC) analysis

Varian 3400 chromatography line, 30 cm in height and 0.32 mm in width, was working with helium as a transporter gas. GC temperature software program Spectra of mass saved in electron ionization (EI) form at 70 eV. The check repetition ranged over a mass of atomic mass units.

#### Statistical design and analysis

The design was a completely randomized block (RCBD) with five replicates. For each treatment, the least significant differences (LSD) were used to test the differences among the means of each parameter.

## Results

### *Populus nigra* leaf extract chemical composition in autumn and spring

The investigated data indicator that there were differences in the chemical structure of *P. nigra* extract between spring and autumn periods under Egypt conditions. It had clear that spring (PLE) extract more enhanced the chemical composition present compared to the autumn extract period. In comparison the autumn (PLE) extract, the spring (PLE) extract contained more tiglic acid, phenol, benzoic acid, dihydrocinnamic acid, cinnamic acid, 4-hydroxyphenylacetic acid, 4-methoxycinnamic acid, 3,4-dimethoxymethyl cinnamate, ferulic acid, caffeic acid, and pinostrobin chalcone as shown on Table 3.

**Table 3:** Chemical composition of *P. nigra* leaves extract during autumn and spring.

Chemical	*P. nigra* leaves	*P. nigra* litter-layer
Heptanal	+	+
Β-eudesmol	+	+
2-Phenylethanol	+	+
Guaiol	+	+
Α-eudesmol	+	+
Γ-selinene	+	+
Δ-cadinene	+	+
Α-elemene	+	+
Γ-cadinene	+	+
1,8-Cineole	+	+
Benzyl alcohol	+	+
Tiglic acid	+	−
Phenol	+	−
Benzoic acid	+	−
1,2-Cyclohexadiol	+	+
1,n-Cyclohexadiol	+	+
Phosphoric acid	+	+
Glycerol	+	+
n-Tricosane	+	+
Pyrocatechol	+	+
Cinnamyl cinnamate	+	+
Succinic acid	+	+
n-Pentacosane	+	+
n-Heptacosane	+	+
Dihydrocinnamic (benzenepropanoic) acid	+	−
Eugenol	+	+
Malic (2-hydroxybutanedioic) acid	+	+
Cinnamic acid	+	−
Protocatechuic aldehyde	+	+
4-Hydroxyphenylacetic acid	+	−
4-Methoxy methyl cinnamate	+	+
Guaiol	+	+
4-Hydroxyhydrocinnamic acid	+	−
4-Methoxycinnamic acid	+	−
3,4-Dimethoxy methyl cinnamate	+	−
Β-Coumaric acid	+	+
3,4-Dimethoxycinnamic acid	+	−
Hexadecanoic acid	+	+
Ferulic acid	+	−
Caffeic acid	+	−
Α-Linolenic acid	+	+
Octadecanoic acid	+	+
Pinostrobin chalcone	+	−
Pinocembrin	+	+
Chrysin (2,5-dihydroxyflavone, mono-TMS)	+	+
5,7-dihydoxy-flavone	+	+
Gallic acid	+	+
Salicine	+	+

### Effect of *P. nigra* spring leaves extract on *C. annuum* growth

The data of (PMMoV) pepper infected had the highest significant decrease mean value of *C. annuum* fruits and growth parameters compared to other treatments. Meanwhile Plant length of health pepper plants treated (25, 50 and 100%) of *Populus* spring leaves extract recorded significantly means value (64.530, 64.040 and 61.528 cm) respectively compared to control while (PMMoV) infected pepper (PMMoV + PLE25 and PMMoV + PLE50%) more significant capable than (PMMoV + PLE100). This side *P. nigra* spring leaf extracts 25% concentration application on health pepper more significantly enhanced than poplar leaves application 50% on pepper number branches as well as (PMMoV + PLE25) the highest significant mean value compared to other infected pepper plants. Fruit weight of pepper plants data had indicator that treated with (PLE 100 and PMMoV + PLE50%) had no significantly mean value between (healthy and infected pepper, respectively. PLE 25 and PMMoV + PLE25 recorded the high mean value compared to other treatment. PMMoV + PLE100% application had enhanced significant mean values compared to PMMoV on the pepper fruit diameter, according to Table 4.

**Table 4:** Effect of foliar application of *P. nigra* spring leaves extract (PLE) and inoculation with pepper mild mottle virus (PMMoV) on morphological characters of pepper plants.

%Treatments	Plant length (cm)	Number of branches	Number of leaves	Fruit weight (g)	Fruit length (cm)	Fruit diameter (cm)
Control	66.824^a^ ± 0.326	8.000^a^ ± 1.730	29.200^a^ ± 1.924	19.792^a^ ± 0.582	5.540^a^ ± 0.371	2.520^a^ ± 0.192
PLE 25	64.530^b^ ± 0.148	7.310^b^ ± 0.517	28.600^abc^ ± 0.112	18.552^b^ ± 0.139	5.140^b^ ± 0.255	2.310^b^ ± 0.153
PLE 50	64.040^c^ ± 0.357	6.810^c^ ± 0.427	28.320^bcd^ ± 0.531	18.132^c^ ± 0.135	4.300^d^ ± 0.169	2.020^cd^ ± 0.145
PLE 100	61.528^f^ ± 0.966	6.310^e^ ± 0.602	27.720^def^ ± 0.530	17.724^e^ ± 0.412	3.426^f^ ± 0.526	1.920^e^ ± 0.150
PMMoV	51.760^h^ ± 1.537	5.800^h^ ± 0.837	27.600^efgh^ ± 1.673	10.140^h^ ± 0.344	2.936^h^ ± 0.244	1.460^h^ ± 0.089
PMMoV + PLE25	63.840^cd^ ± 0.732	6.700^cd^ ± 0.521	28.300^1bcd^ ± 0.352	18.100^ cd^ ± 0.029	4.880^c^ ± 0.285	2.030^c^ ± 0.152
PMMoV + PLE50	62.140^de^ ± 0.376	6.210^f^ ± 0.220	28.050^cde^ ± 0.354	17.420^ef^ ± 0.361	4.204^e^ ± 0.169	1.850^f^ ± 0.125
PMMoV + PLE100	60.772^g^ ± 0.234	6.010^g^ ± 0.472	27.680^efg^ ± 0.101	16.042^g^ ± 0.123	3.028^g^ ± 0.212	1.520^g^ ± 0.317
LSD	0.406	0.359	0.585	0.337	0.088	0.054

Different superscript letters in each row indicate significant differences (*p* < 0.05), ± standard deviation.

### Effect of *Populus* autumn leaves extract on *C. annuum* growth

Data on Table 5 investigated that treated healthy *C. annuum* with autumn leaves extract 100% concentrations of *P. nigra* extract (PLE) had the heights significantly increment mean value plant length flowed with PMMoV + PLE100 compared to other treatments. Pepper branches number (control and PMMoV) treatments had the lowest significantly mean value compared to other treatments. On this side, healthy pepper plants at (PLE 50 and PLE 100%) more enhanced significantly increment mean value (31.800e and 33.400bc) respectively pepper leaves number. On the other hand, pepper fruits weight recorded a significantly increment (28.404 and 25.667 g) at treatment (PLE 100 and PMMoV + PLE100%) respectively while treated with (control and PMMoV) had the lowest significant decrement mean value. Study data showed that treated with (PLE 100, PMMoV + PLE100 and PLE 50%) recorded a significantly increment mean value (7.656, 7.228 and 6.600 cm) respectively of pepper fruit height. The experiment treatments (PMMoV + PLE50, PMMoV + PLE25 and PMMoV) had the lowest significant mean value of pepper fruit diameter.

**Table 5:** Effect of foliar application of *P. nigra* autumn leaves extract (PLE) and plants infected with pepper mild mottle virus (PMMoV) on morphological characters of pepper plants.

%Treatments	Plant length (cm)	Number of branches	Number of leaves	Fruit weight (g)	Fruit length (cm)	Fruit diameter (cm)
Control	66.824^ef^ ± 0.326	8.000^g^ ± 1.730	29.200^gf^ ± 1.924	19.792^f^ ± 0.582	5.540^ g^ ± 0.371	2.520^e^ ± 0.192
PLE 25	68.384^d^ ± 0.468	9.200^e^ ± 0.837	29.600^f^ ± 1.140	21.792^d^ ± 0.789	6.240^de^ ± 0.195	2.620^d^ ± 0.164
PLE 50	69.700^c^ ± 0.897	10.200^c^ ± 0.837	31.800^e^ ± 1.789	25.352^bc^ ± 1.237	6.600^c^ ± 0.158	2.880^c^ ± 0.130
PLE 100	75.068^a^ ± 1.196	11.200^a^ ± 0.837	33.400^bc^ ± 1.140	28.404^a^ ± 0.602	7.656^a^ ± 0.336	3.220^a^ ± 0.109
PMMoV	51.760^h^ ± 1.537	5.800^h^ ± 0.837	27.600^h^ ± 1.673	10.140^h^ ± 0.344	2.936^h^ ± 0.244	1.460^h^ ± 0.089
PMMoV + PLE25	61.940^ g^ ± 0.652	9.200^e^ ± 0.837	32.800^d^ ± 0.837	19.570^fg^ ± 1.018	6.058^f^ ± 0.183	2.300^g^ ± 0.100
PMMoV + PLE50	67.06^e^ ± 1.266	10.200^cd^ ± 0.447	33.600^b^ ± 0.894	20.750^e^ ± 1.342	6.264^d^ ± 0.169	2.440^f^ ± 0.114
PMMoV + PLE100	70.772^b^ ± 1.114	10.800^b^ ± 0.837	34.800^a^ ± 1.303	25.667^b^ ± 0.841	7.228^b^ ± 0.112	2.940^b^ ± 0.207
LSD	0.378	0.359	0.520	0.337	0.088	0.054

Different superscript letters in each row indicate significant differences (*p* < 0.05), ± standard deviation.

### Effect of (PLE) after 20 and 40 days from (PMMoV) on symptoms

Using (SPLE) foliar application with different concentrations, processed infected pepper plants enhanced fruit pepper virus symptoms compared to untreated infected fruit peppers, a regard to Table 6.

**Table 6:** Effect of foliar application of *P. nigra* spring leaves extract (SPLE) after 20 and 40 days from PMMoV inoculation on external symptoms % of pepper plants.

%Treatments	After 20 days		After 40 days				
	mM	M	mM	M	Stunting	Mal	Y
Control	−	−	−	−	−	−	−
PMMoV	+	−	−	+	+	+	+
PMMoV + PLE 25	−	+	+	−	−	+	−
PMMoV + PLE 50	−	−	+	−	−	−	−
PMMoV + PLE 100	−	−	−	−	−	−	−

*mM* mild Mottle, *M* mottle, *St* stunting, *Mal* malformation, *Y* yellowing, − no symptoms.

According to data shown in Table 7, treated infected peppers with 50 and 100 APLE concentrations were more capable of battling virus symptoms of pepper infected peppers when compared to unprocessed infected peppers. Meanwhile, foliar application with (25% APLE is incapable of managing symptoms of (PMMoV).

**Table 7:** Effect of foliar application of autumn *P. nigra* leaves extract (APLE) after after 20 and 40 days from PMMV inoculation on external symptoms of pepper plants.

%Treatments	After 20 days		After 40 days				
	mM	M	mM	M	Stunting	Mal	Y
Control	−	−	−	−	−	−	−
PMMoV	+	−	−	+	+	+	+
PMMoV + PLE 25	−	+	+	+	−	−	−
PMMoV + PLE 50	−	−	+	−	−	−	−
PMMoV + PLE 100	−	−	−	−	−	−	−

*mM* mild mottle, *M* mottle, *St* stunting, *Mal* malformation, *Y* yellowing, − no symptoms.

### Effect of (PLE) after after 20 and 40 days from (PMMoV) on pepper ELISA

Data in Table 8 indicated that treated infected pepper with spring (PLE) foliar application enhanced pepper defense for (PMMoV) compared to untreated. By using (PLE100%) recorded the highest significant decrease in treatment after the first and second. The second season had the same trend. On the other hand, autumn (PLE) applications lead to significantly decreased virus concentration and reproductive.

**Table 8:** Effect of foliar application of *P. nigra* leaves extract (PLE) after first and second from PMMoV inoculation on relative concentration of PMMoV with ELISA of pepper plants.

%Treatments	SPLE	APLE
Control	0.145^e^ ± 0.021	0.422^b^ ± 0.022
PMMoV	0.531^a^ ± 0.041	0.343^c^ ± 0.035
PMMoV + PLE 25	0.401^b^ ± 0.021	0.320^d^ ± 0.030
PMMoV + PLE 50	0.329^c^ ± 0.032	0.343^c^ ± 0.005
PMMoV + PLE 100	0.292^d^ ± 0.035	0.422^b^ ± 0.022
LSD	0.004	0.035

Different superscript letters in each row indicate significant differences (*p* < 0.05), ± standard deviation.

## Discussion

*Populus* leaves turn yellow in autumn, a consequence according to chlorophyll degradation during senescence in response to environmental change^29,30^. The present study showed that concentrations of phenolic acids in leaves were more highly elevated at the beginning of the spring period and decomposed at the end of the active season until leaves maturity^31^. ^32^ found that willow trees’ degraded structure and growth rate turn down on senescence leaves in relation to low leaf water potential. Senescence process leaf damage caused by uncoupled chlorophyll that downstream decreased led to the photosynthesis process not operating and increased chemical decomposition in^33,34^. This concern 75.0% diluted ethanol solvent had a significant effect in extracting phenol composition^35^.

In fact, chlorophyll loss might be a sign of membrane damage, especially if hydrogen peroxide generation is enhanced^36^. By destroying a variety of targets, including proteins, reactive oxygen species can cause significant harm to cell structure and metabolism^37^. In fact, chlorophyll loss might be a sign of membrane damage, especially if hydrogen peroxide generation is enhanced^36^. Reactive oxygen species have the potential to cause significant cell damage. Indeed, phenolic compounds appeared to reduce seedling growth of crop^38^. Meanwhile, phenol structures caught hold of the behaviour of respiratory enzymes. Typically pretentious phenol components are aldolase plus glucose phosphate isomerase, involved in glycolysis and glucose 6-phosphate dehydrogenase^39^.

On the other side, *Populus* is a genus of the Fam. Salicaceae, and included different naturally happening aromatic components such as salicylic acid and salicylic alcohol, as well as aromatic ketones moreover terpenoids furthermore fatty addition to organic acids plus benzyl alcohol also beta-phenyl ethanol, moreover other compounds^40,41^. The researchers also recorded that the majority structures had showed antimicrobial motion, such as salicylic component^42^.

The study present that aspen had different concentrations of deterrent secondary substances relating to bud grow old. This concerning four herbivores had incapable feed selective as defense protection^43–45^. Poplar trees had a surprising occurrence famous as autumn senescence, a vital role in survival trees^46^. The commencement of inception senescence had known altering in metabolic rate of leaf since starting copious photosynthetically active to senescence situation then leaf actively had been incapable of precious components content after that transported out leaf^47,48^. Senescence leaves had more than greater synchronized development in cell organs. It had deconstructed and content reallocated^49^. While the tree cell gets the beginning pointer for preparatory programmed death. Cell dismantled itself then manner initial chlorophyll molecules after that nucleus and mitochondria dismantled^50^.

^51^ indicator that the exogenous function of salicylic acid enhanced the photosynthetic^52^ observed the encouraging consequence of low salicylic acid concentration on increased yield. When cucumber and tomato plants treated with reduced salicylic acid concentrations, fruit output parameters enhanced considerably^53^.

On this concern,^54^ found that foliar application of salicylic acid had a positive effect on early yield and total yield, and that the highest yield occurred in the 0.50 mM salicylic acid treatment. They also suggested that to improve yield, foliar application of salicylic acid had used. On this mention low doses, salicylic acid is more capable of photosynthesis and growth parameters than excessive amounts^55^.

Higher salicylic acid concentrations [10–4 M] inhibited ethylene synthesis, according to^56^. However, the mechanism of action of salicylic acid-mediated ethylene biosynthesis is still unknown. The data had pinpointing the mechanism connected with salicylic acid for ethylene biosynthesis and action will require a lot of debate. ^57^ found chlorophyll reduced following concentrations (100–1 mM) of salicylic acid submission in leaves^58^ reported that virus ability to inhibit or enhance according to salicylic acid dependent signaling.

Salicylic acids enhance the confrontation mechanisms of phytoalexin construction then being capable of cell wall membrane amplification and lignification, furthermore salicylic acid submission on tobacco improved the confrontation with viruses and the resistance movement among plant cells^59^. ^60^ investigated that salicylic acid, an important component, had encouraged endogenous messengers which enhance pathogen resistance. This theory has maintained tobacco plants’ foliar application by salicylic acid, and this improvement persuaded structures of pathogenesis-related proteins and confrontation with the tobacco mosaic virus^61^. This admiration^62^ reported that instruction of virus resistance with phenol stricture is possible. ^63^ found that salicylic acid phytohormone participants in modifiable protection were mostly against abiotrophic moreover hemotrophic pathogens.

The preceding study observed that salicylic acid reduced virus concentrations detected by DAS-ELISA. The diminution in virus concentrations released an enhanced peroxidase enzyme, which is recognized to encourage forming polymerization. It lead to lignin combination plus point had straight connected with an augmented facility of systemically secluded lignin tissues furthermore assist protection from viral infection^64^. This concern salicylic acid excesses production of antioxidants moreover improved virus resistance. Accumulation of salicylic acid following the process had induced in plants challenged by various viruses^65^. An improvement in salicylic acid concentration was essential for plants’ resistance to viral infection and virus replication^65^.

## Conclusion

The experimental data expresses the new applied method using *Populus nigra* leaves extract to increase poplar trees evaluation. Pepper is Egyptian economic and popular crop. Pepper mild mottle virus damaged pepper crops without infect side. The other side *Populus* are deciduous trees and suitable for Egyptian conditions. Ethanol extract of *Populus* leaves analysis recorded high antimicrobial content during spring season. Meanwhile, autumn *Populus* leaves analysis chemical contented before falling. This investigated study effect of different concentrations (25–50 and 100%) as foliar applications of spring and autumn *Populus* leaves individuals on growth parameters of healthy and virus infected pepper. *Populus* leaves extracts foliar applications treated after (20 and 40 days) from pepper transplants. The experimented data recorded spring *Populus* leaves extract had a (negative and positive) significant effect on health and infected pepper growth, respectively on high concentrations. On the other side, autumn foliar *Populus* leaves extracts high concentrations had significant increment on pepper growth and fruits.

## Supplementary Information

Supplementary Information.

## Data Availability

The datasets used and/or analyzed during the current study are available from the corresponding author on reasonable request.

## Abbreviations

PMMoV: Pepper mild mottle virus
PLE: *Populus nigra* leaves extract
SPLE: Spring *Populus nigra* leaves extract
APLE: Autumn *Populus nigra* leaves extract
