# Supplementary material for: Effect of Populus nigra spring and autumn leaves extract on Capsicum annuum infected with pepper mild mottle virus
Source: Sci Rep. 2022 Dec 23;12:22194. doi: 10.1038/s41598-022-24786-2 (PMC9789118; doi:10.1038/s41598-022-24786-2)
Supplement: Supplementary file 1 — Supplementary Information. [file 41598_2022_24786_MOESM1_ESM.doc]

**Material and Methods**

This investigated study highlight the important of *Populus nigra* leaves and indicator differences between chemical strictures during spring and autumn content. In this concern, clear new untraditional process struggle PMMoV infections. During the spring on 2020, the current trial had carried out at Ismailia Governorate, Egypt. After pepper seedlings had three or four true leaves transplanting, Capsicum annuum was cultivated on pots 30 cm in mixed loam and sand (1:1) under greenhouse conditions. Mineral fertilizer had using the nitrogen fertilizer in the structure of calcium nitrate (17% N), potassium fertilizers in the structure of potassium sulphate (48% K_2_O) and phosphorus fertilizers in the structure of phosphoric acid (61.5% P_2_O_5_) according to Tab. (1).To investigate the effect of concentrations (% 25-50 and 100) foliar application of spring Populus nigra leaves extract (SPLE) and autumn Populus leaves extract (APLE) individuals after (20 and 40 days) respectively pepper transplant on growth parameters without infection as a split block treatment. On this concern pepper plant had infected with (PMMoV) during a first week after transplant, then treated at (20 and 40 days) respectively from transplant with foliar application extract, form (SPLE) and (APLE) individual as another split block treatment.

**Table (1). Fertilizer program of *Capsicum annuum* under greenhouse conditions (g/pot)**

| **Week after Transplanting** | **Calcium Nitrate** | **Potassium Sulphate** | **Phosphoric Acid** |
| --- | --- | --- | --- |
| 2 | 0.15 | 0.10 | 0.02 |
| 3 | 0.35 | 0.20 | 0.05 |
| 4 | 0.50 | 0.30 | 0.08 |
| 5 | 0.70 | 0.40 | 0.10 |
| 6 | 1.00 | 0.60 | 0.15 |
| 7 | 1.00 | 0.60 | 0.15 |
| 8 | 1.00 | 0.60 | 0.15 |
| 9 | 1.00 | 0.60 | 0.15 |
| 10 | 0.70 | 0.40 | 0.10 |
| 11 | 0.70 | 0.40 | 0.10 |
| 12 | 0.70 | 0.40 | 0.10 |
| 13 | 0.70 | 0.40 | 0.10 |

**Greenhouse conditions**

Greenhouse air was 21-30 ºC during daylight and 16-18 ºC at nighttime. Atmosphere had relative humidity from 40 to 90%. The suggested vapor pressure deficit (VPD) might be from 3 to 7 g/m^3^ **[17], [18], [19] and [20]**. The solar radiation between 200-450 W/m^2^ within the gable-even-span greenhouse was 6 measured and evidenced for short and long requisites. In contrast, other greenhouses conditions control using some apparatus, like black net sheets and natural airing systems **[20]**.

**Tree Materials**

    Collection of tree material poplar tree spring leaf collected in March 2019 and 2020 from trees growing in the nursery of timber trees department at the Horticulture Research Institute Agriculture Research Center, Giza, Egypt. Senescent leaves were collected in September (2018 and 2019). The samples dried in the electric oven at 40˚C until they reached constant weight, according to **[21].** Dried material was ground by an electric mixer to find the crush forms of each sample. The powder preserved in sterilised glass jars.

**Preparation extracts samples:**

    Then samples were air dried in the laboratory for seven days under room conditions and later in the electric oven for two days at 40˚C **[22].** The dried material then pulverised using a blender (electric mixer) to get powder forms of each sample. The powder collected and kept in clean and sterile conditions. Each leaf in spring and autumn, an individual dried powdered sample 500g had lain in a 2000 ml beaker and processed by drenching 1000 ml of ethanol solvent. Then they enclosed with aluminium foil and put into a water bath 60˚C and had shaken to get homogenous solutions. After that, the samples filtered and evaporated by a rotary evaporator 60˚C to isolate the solvent extract and store it in clean-capped glass bottles and reserve it in the refrigerator for reuse **[23].**

**Source of virus isolates:**

Several field visits had been conducting to pepper plant growing areas in the Ismailia Governorate. The naturally infected pepper plants contain viral symptoms including mottling, leaf distortion, yellowing, and stunting collected according to Fig (1). After being collected from the field, the infected leaf samples had placed in cool boxes and stored at 80 °C for later use.


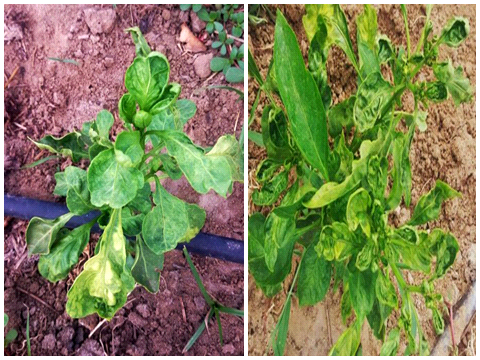


**Fig. 1: The naturally infected pepper plant had indicator symptoms including mottling, leaf distortion, and stunting in the field.**

**Mechanical inoculation:**

Mechanical inoculation carried out according **[24].** Infectious sap extracted from young leaves indicator clear and typical symptoms. Leaves were ground in a sterilized mortar with a few drops of 0.2M phosphate buffer. The extracted sap filtrated through two layers of chasse cloth and then centrifuged for 5 min. at 5000 rpm. Before it was used to inoculate the leaves of tested plant seedlings. The infected plant extract measured on a NanoDrop device and found that the virus concentration was high.

**Propagation of virus isolates:**

     Infected leaf samples were ground in a phosphate buffer solution (pH7.2). Infectious sap had mechanically inoculated onto *Chenopodium amaranticolor*. The single local lesion assay used for biological purification of the isolate and propagated on healthy pepper plants.

**Virus identification:**

**Host range and symptomatology:**

Twenty-one plant species belonging to four families mechanically inoculated with infectious crude sap expressed from pepper plants. The seedlings of each host species inoculated and observed daily for symptom development, and the mechanically inoculated plants kept under observation in insect-proof cages under the greenhouse. Three weeks later, plants examined visually for any signs of symptom appearance. Symptomless plants were checked for virus infection by back inoculation of *Chanopodium amaranticolor* leaves and/or the ELISA technique by **[25].**

Different symptoms observed on the infected pepper plants. This virus infection had indicator mottling, leaf distortion, yellowing, and stunting. This virus propagated on pepper plants, which developed the same symptoms as those in naturally infected plants. This isolated of PMMoV indicator different styles of symptoms on pepper hosts, such as mild mottling, mottling, yellowing, and malformation (Table 1 and Fig. 2). The incidence of PMMoV confirmed by back inoculation with *Chanopodium amaranticolor*. The tested plants could divide, according to their reactions, into two groups:

**Susceptible hosts to PMMoV.**

a- **Plants reacted with systemic symptoms.**                                                              .
Systemic symptoms observed in the tested *Capsicum annum* L. cv. California, *Capsicum fratescens* L. cv. Chilli and *Nicotiana clevelandii* Fig. (2). Systemic symptoms, general appear nearly 11–14 days after inoculation..

**b- Plants reacted with local lesions**. .
   Virus isolate produced chlorotic local lesions on the inoculated leaves of *Chanopodium amaranticolor*, Ch. quinoa and necrotic local lesions on the inoculated leaves of *Datura metal*, *Datura stramonium*, *Nicotiana tabacum,* and *N. glutinosa* nearly 7–10 days after inoculation (Fig. 2).

**2-Unsusceptible plants.**

These plant species were not susceptible to pepper infection. These plants belong to different families: Cucurbitaceae, Fabaceae, and *N. arusica*. Host range studies for diagnosis will usually be most useful for those infecting a relatively narrow range of plants **[26].**

         The general outlook of the result in table (2) indicator that the studied isolate of PMMoV had a wide host range between members of the family Solanaceae. On the other side, the virus infects a few species of Chenopodiaceae. PMMoV induced mottling, yellowing, and malformation symptoms in the family Solanaceae. The informed data in table (1) confirmed the results of **[27].**

**Tab. (2): The reaction of different hosts to Pepper mild mottle virus**.

| **Family** | **Host plant** | **Symptoms** |
| --- | --- | --- |
| *Chenopodiaceae* | *Ch. amaranticolor* Coste and Reyn  *Ch. quinoa* Wild  *Beta vulgaris* | CLL  CLL NS |
| *Cucurbitaceae* | *Cucurbita pepo* cv. Cavili  *Cucurbita pepo* cv. Eskandarni  *Cu. maxima* cv. Wintersquash  *Cucumis sativus* cv. Balady  *Citrullus lanatus* cv. Giza 2 | NS  NS  NS  NS NS |
| *Fabaceae* | *Glycine max* L.cv.Giza22  *Lupinus termis* cv. Lupine  *Phaseolus vulgaris* cv. Giza 4  *Pisium sativum* L. cv. Sugar sweet  *Vicia faba* cv. Giza 3 | NS  NS  NS  NS NS |
| *Solanaceae* | *Capsicum annum* L. cv. California  *Capsicum fratescens* L.cv. Chilli  *Datura metal*  *Datura stramonium*  *Nicotiana tabacum* L.cv.Whit Burley | M+Y  M+Mf  NLL  NLL  NLL |


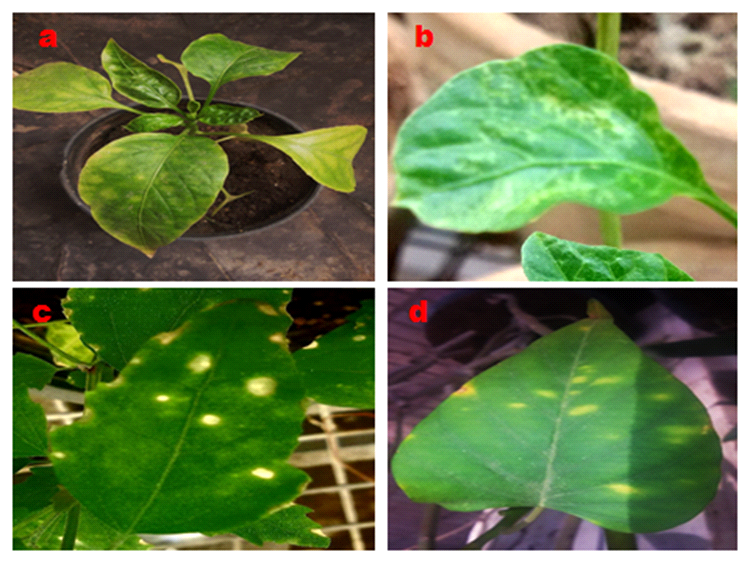


**Fig. 2: Symptomology of PMMoV (a) mottling and yellowing on *Capsicum annum* L. cultivar California,b) mottling on *Capsicum fratescens* L.cv. Chilli(c) Local chlorotic lesions on *Chanopodium amaranticolor* (d) Local necrotic lesions on *N. glutinosa***

**Modes of transmission:**

Mechanical transmission.

  Inoculums prepared by homogenising infected pepper leaves with a few drops of phosphate buffer (pH 7.2) in a sterilised mortar. Leaves of host plants previously dusted with carborundum (600 mech) rubbed with the forefinger or with a cheesecloth pad previously soaked in the inoculum. The plants rinsed with tap water and kept in the insect proof greenhouse. Obtainment results revealed that PMMoV easily transmitted mechanically to indicator hosts like *Chenopodium amaranticolor* which indicator chlorotic local lesions..

**Insect transmission.**

 Two aphid species, name, *Aphis faba* (scop) and *Myzus persicae* (sulz) checked for their ability to transmit the isolated virus. *A. faba* (scop) and *M. persicae* (sulz) maintained on virus- free health faba beans for *A. faba* (scop) and cabbage plants for *M. persicae* (sulz) and kept under insect-proof cages in the greenhouse. The aphids starved for one hour and then transferred to feeding for a 30 minute acquisition feeding period on diseased pepper plants. At the end of the feeding period, aphids transferred to healthy plants at a rate of 10 aphids/plant. After a 24 hour feeding period, the insects had killed by spraying all tested plants with an effective insecticide (malathion 0.2%). Symptoms and the percentage of transmission recorded.

Results indicator these *A. faba* (scop) and *M. persicae* (sulz) did not able to transmit the virus. None of the tested plants produced any symptoms.

**Seed transmission of virus:**

To study the transmission of (PMMoV) through seeds. Two hundred pepper seeds cv. California collected from previously inoculated infected peppers had sown in 20 cm sterilized pots and kept in an insect- proof greenhouse for symptom observation for three weeks after sowing, and the percentage of seed transmission calculated. (PMMoV) transmitted through pepper seeds. Data showed that the percentage of seed transmission differed according to cultivar. (PMMoV) transmitted at 38%. The result confirmed using ELISA.

**Molecular characterization:**

**RNA extraction.**

RNA extraction from leaf samples carried out using the RNeasy Plant Mini Kit (QIAGEN) according to the manufacturers’ instructions.

**Primers for the coat protein gene of (PMMoV):**

 For the amplification of the capsid protein (CP) gene (474 bp), two pairs of specific primers (CP/s: 5′-ATGGCATACACAGTTACCAGT-3′) and (CP/a: 5′-TTAAGGAGTTGTAGCCACACGTA3′) used in RT-PCR **[28]**.

**One-step RT-PCR:**

One-step RT-PCR reactions carried out using the “iScript One Step qRT-PCR Kit” (BIOMATIK) in a 25 µL reaction volume. Each reaction contained 1 µL of the RNA extract (40 mg of total RNA), 12.5 µLi Green Mastermix, 1.5 µL of 10 µM of each primer, 0.5 µL of qRT-PCR Enzyme Mix, and 25 μL of nuclease-free water. Synthesis of cDNA done at 42°C for 30 min and denaturation at 95°C for 10 minutes, followed by 35 cycles of 94°C for 30 sec, 50°C for 1 min, 72°C for 1 min, and a final cycle of 72°C for 10 min **Velasco *et al*. (2011)**. 5 μL of PCR products were loaded into 1% agarose gels with a 100 bp DNA ladder (BIOMATIK) and pictures taken under UV light with a digital imaging system gel doc (Syngene Bio Imagins, IN Genius).

**Analysis of RT-PCR products:**

The *cp* genes of PMMoV collected from Ismailia had isolated using RT-PCR with specific primers. The PMMoV-*cp* gene had (~474) bp as shown in (Fig.3).


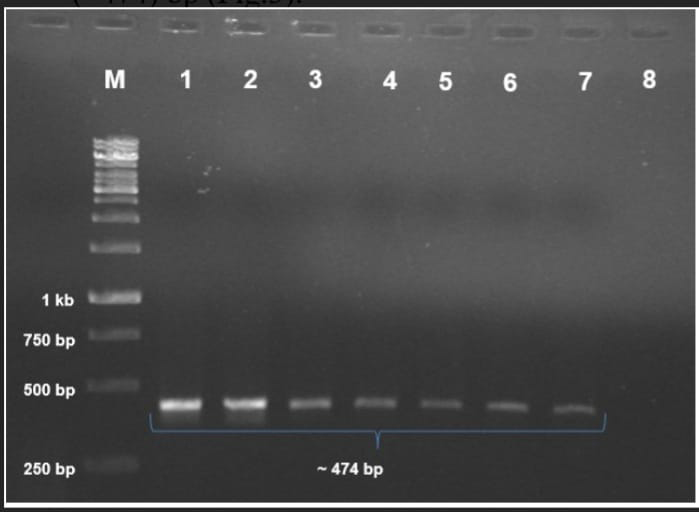


**Fig. 3: Agarose gel electrophoresis of RT-PCR amplified products. M: 1 kb DNA ladder (Promega); 1–7: seven infected pepper samples; 8: a negative sample**

**Experimental design and treatments:**

A randomized complete block design used with fourteen treatments, everyone had sixteen plants, including control. It had contained four replicated. Each one contained four pots had one plant per pot. Pepper seedlings treated with extract by spraying whole leaves, even run-off, with different (PLE) concentrations after 20 and 40 days, respectively, follows:

1-pepper seedling untreated control.

2- Pepper seedlings had foliar with 25% (SPLE) +75% tap water.

3- Pepper seedlings had foliar with 50% (SPLE) + 50% tap water.

4- Pepper seedlings had foliar with 100% (SPE).

5- Pepper seedlings had foliar with 25% (APLE) +75% tap water.

6- Pepper seedlings had foliar with 50% (APLE) +50% tap water

7- Pepper seedlings had foliar with 100% (APLE).

8- Pepper seedling infected (PMMoV) without foliar application.

9- Pepper seedlings infected (PMMoV) foliar with treated with 25% (SPLE) +75% tap water.

10- Pepper seedlings infected (PMMoV) foliar with 50% (SPLE) +50% tap water.

11- Pepper seedlings infected (PMMoV) foliar with 100% (SPLE).

12- Pepper seedlings infected (PMMoV) foliar with 25% (APLE) +75% tap water.

13- Pepper seedlings infected (PMMoV) foliar with 50% (APLE) +50% tap water.

14- Pepper seedlings infected (PMMoV) foliar with 100% (APLE).

**Gas chromatography (GC) analysis:**

Varian 3400 chromatography line, 30 cm in height and 0.32 mm in width, was working with helium as a transporter gas. GC temperature software program Spectra of mass saved in electron ionization (EI) form at 70 eV. The check repetition ranged over a mass of atomic mass units.

**Statistical design and analysis:** .
 The design was a completely randomized block (RCBD) with five replicates. For each treatment, the least significant differences (LSD) were used to test the differences among the means of each parameter.
